# Astaxanthin as a potential therapeutic agent in polycystic ovary syndrome: a review of its antioxidant, anti-inflammatory, and metabolic mechanisms

**DOI:** 10.3389/frph.2026.1901207

**Published:** 2026-07-13

**Authors:** Jialai Wang, Ying Yang, Ziwei Wang, Chengdong Liu, Yanhua Han, Xiaoke Wu, Hui Chang

**Affiliations:** 1 The First Clinical Medical College, Heilongjiang University of Chinese Medicine, Harbin, China; 2 Department of Traditional Chinese Medicine, Affiliated Hospital of Jiangsu University, Zhenjiang, China; 3 Department of Gynecology, First Affiliated Hospital of Heilongjiang University of Chinese Medicine, Harbin, China

**Keywords:** anti-apoptotic, anti-inflammatory, antioxidative stress, astaxanthin, polycystic ovary syndrome

## Abstract

Polycystic ovary syndrome (PCOS) is the most common heterogeneous disorder among women of reproductive age, characterized by hyperandrogenism, ovulatory dysfunction, and polycystic ovarian morphological changes as its core clinical features. This disease severely affects women's reproductive health and significantly increases the risk of metabolic complications. The pathogenesis of PCOS is complex, involving various pathophysiological mechanisms such as oxidative stress, chronic low-grade inflammation, and insulin resistance. Currently, lifestyle interventions and first-line pharmacological treatments are the primary clinical strategies for managing PCOS. However, existing approaches face numerous limitations in terms of efficacy, safety, and patient compliance, including incomplete therapeutic effects, drug-related side effects, poor adherence, and a lack of long-term safety data, which necessitate further optimization and breakthroughs. Natural compounds have been widely utilized as therapeutic agents worldwide, with some showing potential advantages in preclinical and pharmacological studies, positioning them as potential alternatives to modern drugs. In recent years, astaxanthin, a natural compound, has garnered attention for its auxiliary effects in the treatment of PCOS due to its potent antioxidant and anti-inflammatory properties. Astaxanthin, a strong natural antioxidant derived from Haematococcus pluvialis, exhibits significant antioxidant, anti-inflammatory, anti-proliferative, and anti-apoptotic activities. To systematically evaluate the therapeutic potential of astaxanthin for PCOS, this study conducted a systematic review in strict accordance with the PICOS principles. A comprehensive literature search was performed across PubMed, Web of Science, and Scopus for relevant studies published between January 2020 and March 2026. The retrieved records were screened based on predefined inclusion and exclusion criteria, and the finally included studies were subjected to mechanistic analysis. This review provides a comprehensive analysis of the potential mechanisms by which astaxanthin, as a dietary supplement, improves PCOS through various pathways, including enhancing insulin sensitivity, activating the Nrf2 antioxidant pathway, inhibiting the NF-κB inflammatory signaling pathway, and modulating cellular apoptosis. Furthermore, it delineates the limitations and therapeutic prospects of astaxanthin supplementation, clarifying its significant value in the adjuvant treatment of PCOS and highlighting the key issues that warrant further investigation.

## 1 Introduction

Polycystic ovary syndrome (PCOS) is one of the most common endocrine and metabolic disorders among women of reproductive age, affecting approximately10% of women of childbearing age. It is clinically characterized by hyperandrogenemia, infrequent or absent ovulation, and polycystic ovarian changes, making it a common cause of female infertility and resulting in significant health and economic burdens (1). In addition, women with PCOS face a higher risk of metabolic complications, such as type 2 diabetes, dyslipidemia, and cardiovascular diseases (2). On one hand, the pathogenesis of PCOS remains unclear. External factors involve environmental pollution, life stress, and diet, while internal factors are associated with oxidative stress (OS), mitochondrial dysfunction,chronic low-grade inflammation, and metabolic disturbances that impair normal ovarian function (3). On the other hand, due to the heterogeneity of the PCOS population and inherent racial/ethnic differences, existing diagnostic criteria are difficult to apply universally, which further impedes the development of targeted treatment strategies (4). In terms of treatment, lifestyle interventions and pharmacotherapy are the core approaches for managing PCOS. Lifestyle modifications focus on balanced nutrition and regular exercise, aiming to control body weight, improve metabolism, and reduce the risk of related complications. In pharmacotherapy, metformin and certain thiazolidinediones are considered the first-line medications for PCOS management; however, drug treatments carry various side effects, and their efficacy is controversial in patients who are neither insulin-resistant nor obese (5, 6). Therefore, developing a new, safe, and effective symptom relief strategy is extremely urgent.

In recent years, natural products have become a research focus in the health management of PCOS due to their unique bioactivities and pharmacological effects, including anti-inflammatory, antioxidative, anticancer, and antidiabetic properties (7).

**Figure 1:**
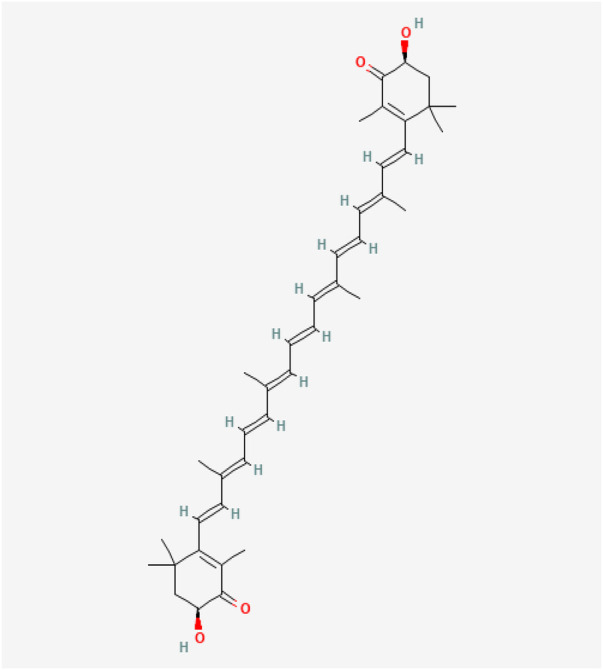
Astaxanthin (3,3'-dihydroxy-β-carotene-4,4'-dione), https://pubchem.ncbi.nlm.nih.gov/compound/Astaxanthin.

Astaxanthin, chemically named 3,3'-dihydroxy-β-carotene-4,4'-dione, is a natural carotenoid compound widely distributed in various microorganisms and seafood (Figure 1). It possesses remarkable antioxidant, anti-inflammatory, antiproliferative and anti-apoptotic properties. Its oxygen radical absorbance capacity (ORAC) is 100–500 times higher than that of β-tocopherol, and its free radical inhibitory activity is 10 times that of related antioxidants such as α-tocopherol and α-carotene (8) and has been approved for use as a dietary supplement in the United States (9). Its molecular structure includes 13 conjugated double bonds as well as hydroxyl and keto groups at the terminal rings, which collectively endow astaxanthin with potent antioxidant activity, enabling it to effectively neutralize reactive oxygen species, scavenge free radicals, and quench singlet oxygen. This helps alleviate oxidative stress-induced cellular damage and contributes to maintaining overall health (10, 11). In PCOS, oxidative stress is involved in the development of multiple pathological processes, including abnormal follicular development, insulin resistance, and hyperandrogenism (12). Clinical studies indicate that astaxanthin may serve as a potential adjuvant therapy for PCOS, primarily improving symptoms by reducing oxidative stress and inflammatory responses (13). Mechanistically, astaxanthin can activate key antioxidant signaling pathways such as Nrf2/PGC-1α and MAPK/PI3K/AKT, thereby promoting the production of antioxidant enzymes including SOD, GST, GPX, and catalase. Simultaneously, it can inhibit the production of pro-inflammatory cytokines by blocking NFκB or JAK/STAT pathways (14, 15). Through these multiple protective mechanisms, astaxanthin provides comprehensive antioxidant defense to cells and intervenes in the core pathophysiological processes of PCOS, thereby potentially improving reproductive function in patients. This article aims to explore the mechanisms by which astaxanthin improves PCOS, providing a theoretical basis for its clinical application.

## 2 Method

### 2.1 Search strategy

To conduct a comprehensive systematic literature search, controlled vocabulary and free text terms were used in conjunction with the Boolean operators “AND” and “OR”. The following text words: (“Polycystic Ovary Syndrome” OR “PCOS” OR “Stein-Leventhal Syndrome”) AND (“Astaxanthin” OR “ASTX” OR “Haematococcus pluvialis” OR “Carotenoid”) AND (“Insulin Resistance” OR “Oxidative Stress” OR “Inflammation” OR “Oocyte Quality” OR “Embryo Outcome”) appeared in all fields of the articles. Multiple databases, including PubMed, Scopus, and Web of Science, were searched for articles published in English from January 2020 to March 2026.

### 2.2 Inclusion and exclusion criteria

A study was included if it met all of the following inclusion criteria: (1) published paper had full length and was in a peer-reviewed source; (2) the study participants were human subjects (clinical studies) or mammalian *in vivo* animal models (preclinical studies); (3) the intervention involved Astaxanthin, either as monotherapy or part of a combination therapy; (4) the route of administration included oral, injectable, or topical application; and the outcomes assessed were PCOS-related core indicators (including metabolic parameters, hormonal levels, oxidative stress biomarkers, and pathological features such as ovulation rates), providing qualitative or quantitative data before and after the intervention.

Studies were excluded if they met any of the following criteria: (1) publication in non-peer-reviewed sources (e.g., preprint servers, news reports); (2) appearance only in abstract form without access to the full text; pure *in vitro* cell line studies (unless serving as supplementary mechanistic evidence); (3) interventions containing additional bioactive ingredients (e.g., specific glucose-lowering drugs or hormones) with primary therapeutic effects where the independent effect of antioxidants could not be isolated; (4) studies irrelevant to the pathophysiological mechanisms or treatment of PCOS; (5) duplicate publications.

## 3 Mechanisms of astaxanthin's antioxidant and anti-inflammatory effects

### 3.1 Activation of the Nrf2 pathway to enhance antioxidant defense

Oxidative stress refers to a state in which there is an imbalance between oxidants and antioxidants in the body, leading to excessive accumulation of reactive oxygen species (ROS). Numerous studies have shown that levels of oxidative stress markers in patients with PCOS are significantly higher than those in healthy individuals, and this excessive oxidative stress can severely impair follicular function (16). Cells possess an intrinsic antioxidant defense system composed of various enzymes, among which superoxide dismutase (SOD) and glutathione peroxidase (GSH-Px) can eliminate ROS generated during cellular metabolism, thereby maintaining ROS homeostasis under physiological conditions (17).

The core antioxidant mechanism of astaxanthin is closely related to its activation of the Nrf2/HO-1 signaling pathway, which promotes the transcriptional expression of multiple antioxidant target genes (18). The Nrf2 signaling pathway plays a central role in maintaining the body's redox balance; upon activation, Nrf2 translocates to the nucleus, initiating the expression of antioxidant enzymes, thereby alleviating oxidative stress and protecting cells from damage (19). In a study of granulosa cells (GCs) derived from healthy women aged 20–38 years, astaxanthin was found to significantly reduce oxidative stress-induced ROS levels (*P* < 0.01) and cell death rate (*P* < 0.05). Its mechanism of action mainly involves the inhibition of KEAP1, promotion of NRF2 nuclear translocation, and activation of downstream antioxidant enzyme systems, suggesting the potential application of astaxanthin in assisted reproductive technology (20). A randomized controlled study by Roghaye Gharaei et al. (21) further demonstrated that administration of astaxanthin significantly increased serum catalase (CAT) and total antioxidant capacity (TAC) levels, enhancing overall antioxidant capacity and SOD activity, thus reducing oxidative stress in the body. This effect is closely associated with the upregulation of Nrf2, heme oxygenase-1 (HO-1), and NAD(P)H quinone oxidoreductase 1 (NQO1) expression in GCs. Astaxanthin significantly enhances endogenous antioxidant defense by activating the Nrf2/HO-1 pathway, effectively neutralizing free radicals and protecting cell membrane integrity. In addition, preclinical studies have shown that in a PCOS rat model, high-dose astaxanthin combined with omega-3 fatty acids and metformin improved endometrial receptivity. This effect may be related to the reduction of oxidative stress and the regulation of key implantation biomarkers, suggesting that this combination therapy may hold promise for improving fertility outcomes in women with PCOS (22). Furthermore, a meta-analysis of clinical trials on astaxanthin supplementation indicated that astaxanthin significantly improved oocyte maturation rates and total antioxidant capacity in follicular fluid. The underlying mechanism lies in astaxanthin's ability to reduce oxidative stress and enhance antioxidant defenses, thereby improving the ovarian microenvironment (23).

In summary, accumulating evidence indicates that the activation of the Nrf2/HO-1 signaling pathway constitutes the core mechanism by which astaxanthin ameliorates PCOS. This pathway upregulates the expression of key antioxidant enzymes, specifically SOD and CAT, to effectively scavenge excess ROS and alleviate oxidative damage to granulosa cells. Additionally, it enhances endometrial receptivity through synergy with omega-3 fatty acids and metformin. Evidence spanning multiple levels confirms that astaxanthin strengthens the endogenous antioxidant defense system, optimizes the ovarian microenvironment, and ultimately improves oocyte quality and fertility outcomes. Therefore, targeted antioxidant therapy against the Nrf2 pathway, particularly nutritional interventions featuring astaxanthin, offers a highly promising novel adjuvant strategy for the management of PCOS.

### 3.2 Inhibition of the NF-κB key signaling nexus and improvement of the reproductive microenvironment

Chronic low-grade inflammation is another important characteristic of PCOS. Astaxanthin can block the expression of downstream pro-inflammatory factors TNF-α, IL-6, IL-1β, COX-2, iNOS, and MMPs by inhibiting the degradation of IκB-α and the nuclear translocation of NF-κB (24). Clinical studies have shown that Administration of astaxanthin significantly reduces serum levels of MDA, IL-6, IL-1β, and TNF-α in patients with PCOS, alongside improvements in the number of oocytes retrieved, the number of mature oocytes, and the rate of high-quality embryos (25, 26). A randomized controlled pilot trial involving 44 patients with PCOS at high risk of ovarian hyperstimulation syndrome demonstrated that astaxanthin intervention downregulated the expression of the receptor for advanced glycation end products in granulosa cells, reduced the p-IκB/IκB ratio, and decreased interleukin-6 levels in follicular fluid, while vascular endothelial growth factor showed a downward trend. These findings suggest that astaxanthin may improve controlled ovarian stimulation outcomes in this population through anti-inflammatory and anti-stress pathways (27).

The combined use of astaxanthin and curcumin in PCOS models demonstrates synergistic anti-inflammatory and antioxidant effects: ovarian CAT activity is significantly increased, IFN-*γ* and IL-6 levels are markedly reduced, while the number of oocytes increases and the rate of abnormal oocytes decreases, providing better therapeutic outcomes than monotherapy. This combination strategy alleviates systemic and local oxidative stress and inflammatory damage, thereby promoting follicular development and ovulation (28). Astaxanthin has the ability to cross the blood-brain barrier, allowing it to regulate hypothalamic inflammation. Randomized controlled studies indicate that the astaxanthin treatment group exhibits reduced expression of aging-related proteins such as p16, p21, and pro-inflammatory factors, which helps restore the function of the hypothalamic-pituitary-ovarian (HPO) axis (28). Animal experiments further confirm that astaxanthin can significantly reduce the expression of inflammatory factors such as TNF-α, IL-6, and NF-κB in the ovarian and liver tissues of PCOS rats. Studies by Toktay et al. (29) and Tuğba (30) examined the therapeutic effects of astaxanthin on ovarian and liver tissue inflammation in PCOS rats. Their findings indicate that astaxanthin treatment reduces the expression of TNF-α, IL-6, and NF-κB in ovarian and liver tissues. Among the doses tested, 200 mg/kg produced the most significant effects, elevating serum FSH levels, and reducing body weight, ovarian weight and volume, the number of cystic follicles, as well as serum levels of LH, testosterone (T), PGE2, IL-17, TNF-α, and IL-18. Mechanistic studies reveal that astaxanthin upregulates Klotho expression and inhibits the Wnt/β-catenin and MEK/ERK signaling pathways, thereby suppressing inflammatory responses, improving hormone levels, reducing cystic follicles, and ultimately alleviating PCOS symptoms (31). Current evidence indicates that astaxanthin demonstrates multifaceted anti-inflammatory potential in the treatment of PCOS. It exerts multi-target therapeutic effects by inhibiting the NF-κB pathway, modulating the hypothalamic-pituitary-ovarian (HPO) axis, and attenuating central-peripheral inflammation, thereby alleviating chronic inflammation and endocrine dysregulation, and offering a novel perspective for clinical intervention.

## 4 Astaxanthin improves insulin sensitivity and glucose-lipid metabolism

### 4.1 Improvement of insulin sensitivity and glucose metabolic homeostasis

Insulin resistance (IR) is one of the core pathological changes in PCOS and is often accompanied by compensatory hyperinsulinemia. Oxidative stress is a key driving factor in the development of IR, exacerbating glucose metabolism disorders by impairing insulin signaling pathways and β-cell function (17). Astaxanthin, as one of the most potent natural antioxidants known to date, exhibits significantly superior antioxidant activity compared to common antioxidants such as vitamin C, vitamin E, coenzyme Q10, and α-lipoic acid (15), showing considerable potential in improving insulin sensitivity and glucose metabolism.

Studies indicate that astaxanthin can improve glucose metabolism abnormalities in PCOS patients through multiple mechanisms. Clinical research by Masaharu Urakaze et al. (32) found that after 12 weeks of continuous Administration of astaxanthin, PCOS patients showed a significant reduction in fasting blood glucose levels, along with a marked decrease in serum immunoreactive insulin levels compared to baseline, suggesting improvements in insulin secretory function and sensitivity. At the molecular level, astaxanthin can upregulate the expression of glucose transporter 4 (GLUT4), promoting glucose uptake in peripheral tissues and improving β-cell function, thereby alleviating hyperglycemia and insulin resistance. Animal experiments by Nishida et al. (33) further confirmed that astaxanthin activates the AMP-activated protein kinase (AMPK) signaling pathway, stimulates mitochondrial biogenesis in skeletal muscle, and significantly improves hepatic insulin resistance in obese mouse models. Moreover, cell studies by Chunmei Li et al (34) found that astaxanthin can enhance insulin signaling by inhibiting S6K1 activity, promoting glucose uptake in adipocytes and myotubes, providing evidence that astaxanthin increases glucose uptake by promoting GLUT4 translocation.

A clinical study involving 92 PCOS patients demonstrated that after 3 months of Administration of astaxanthin, patients exhibited significant reductions in body mass index (BMI), anti-Müllerian hormone (AMH), fasting insulin, HOMA-IR index, basal luteinizing hormone (bLH), and basal testosterone (bT) levels compared to pre-treatment values. These findings suggest that astaxanthin not only helps improve insulin resistance but also aids in restoring hormonal balance, thereby enhancing embryo quality and improving pregnancy outcomes (35). Additionally, the combined application of astaxanthin and metformin shows synergistic therapeutic potential: metformin primarily improves insulin sensitivity, while astaxanthin mitigates ovarian oxidative stress damage through its strong antioxidant capacity, collectively exerting a protective effect (29). Current evidence indicates that astaxanthin, through mechanisms including enhancing antioxidant defenses and regulating GLUT4 and AMPK pathways, holds significant potential in improving insulin resistance and glucose metabolism disorders associated with PCOS.

### 4.2 Regulation of lipid metabolism

Abnormal lipid metabolism is a common complication of PCOS, manifested as visceral fat accumulation, hypertriglyceridemia, and non-alcoholic fatty liver disease (NAFLD), among others. These abnormalities exacerbate insulin resistance, which in turn promotes local ovarian hyperandrogen production, forming a vicious cycle. Studies have shown that astaxanthin can effectively regulate lipid metabolism through multi-target mechanisms, thereby intervening in this cycle.

The core role of astaxanthin in improving lipid metabolism lies in its direct regulation of hepatic lipid synthesis and breakdown. Multiple animal experiments have confirmed that astaxanthin can significantly reduce liver lipid accumulation induced by a high-fat diet (HFD) and decrease levels of total cholesterol (TC), triglycerides (TG), and low-density lipoprotein cholesterol (LDL-C) in serum (36). At the molecular level, astaxanthin inhibits lipid formation at the source by activating the Nrf2 pathway and suppressing the expression of sterol regulatory element-binding protein-1c (SREBP-1c) and its downstream target fatty acid synthase (FASN) (37, 38). Xie et al (39) found that supplementation with 60 μM astaxanthin could regulate sbp-1/mdt-15 and the insulin/IGF-1 signaling pathway, downregulate fat-6/fat-7 expression, significantly reduce overall fat deposition and triglyceride levels, and reverse lipid droplet accumulation, thereby effectively modulating lipid metabolism. It is noteworthy that the regulatory effect of astaxanthin on lipid metabolism is reversible (40); its improvement effect diminishes after discontinuation, suggesting that continuous supplementation may be required for long-term management of PCOS.

Ferroptosis is a non-apoptotic cell death mechanism characterized by iron-dependent membrane lipid peroxidation (41). Leveraging its potent membrane antioxidant capacity, astaxanthin alleviates hepatic lipid deposition and cellular injury, effectively suppresses ferroptosis, and ameliorates dysregulated lipid metabolism, thereby offering a novel target for improving reproductive outcomes in PCOS (42). Iron serves as a crucial mineral that regulates female physiological functions and tumorigenesis, playing a central role in maintaining ovarian homeostasis. Dysregulated ferroptosis in ovarian GCs directly exacerbates ovarian dysfunction and critically contributes to the pathogenesis of PCOS (43). Further investigation Huang Liu et al. utilizing network pharmacology, *in vitro* cellular experiments, and *in vivo* animal models, demonstrated that astaxanthin significantly enhances sperm concentration and progressive motility in a mouse model of oligoasthenozoospermia. Mechanistically, this effect is associated with the reduction of ACSL3, MDA, and ferrous ion levels, alongside the upregulation of Steap3, VDAC, GPX4, GLS2, GSH-Px, and FADS2 expression. These actions suggest that astaxanthin promotes spermatogenesis by inhibiting ferroptosis, modulating fatty acid metabolism, and alleviating mitochondrial oxidative stress damage (44). Collectively, these effects improve the metabolic foundation in PCOS patients, providing important pathophysiological insights for alleviating insulin resistance and hyperandrogenemia.

In summary, astaxanthin intervenes in the dysregulated lipid metabolism associated with PCOS through multi-target mechanisms. Its core effects involve suppressing hepatic lipogenesis via the Nrf2/SREBP-1c pathway and blocking ferroptosis through its unique membrane antioxidant capacity. This dual action effectively disrupts the vicious cycle linking dyslipidemia, insulin resistance, and hyperandrogenism. Although these lipid-modulating effects may diminish upon discontinuation, this observation underscores the potential utility of astaxanthin in long-term metabolic management. Collectively, these findings establish astaxanthin as a novel adjuvant therapy that addresses the complex metabolic pathology of PCOS by concurrently improving lipid homeostasis and inhibiting cellular ferroptosis.

## 5 Protective effects of astaxanthin on ovarian function

### 5.1 Activation of downstream PI3K/Akt signaling pathway to regulate apoptosis

An imbalance in cell death/anti-apoptotic signaling is considered a major pathogenic mechanism of PCOS. In addition to its potent free radical-scavenging capability, astaxanthin exhibits significant anti-apoptotic properties, partly related to its regulation of Nrf2 and its downstream target genes (16). In PCOS, ROS not only contribute to disease development but also play a key role in follicle and granulosa cell apoptosis.

Animal studies by Yang et al. (45) found that supplementation with 5 μmol/L astaxanthin effectively enhanced cell viability and proliferation, reduced ROS levels, decreased cell apoptosis, increased mitochondrial membrane potential, and promoted the synthesis and secretion of steroid hormones. Research indicates that astaxanthin can significantly reduce oxidative stress, thereby lowering cell death rates. Its mechanism is associated with the activation of the phosphoinositide 3-kinase/protein kinase B (PI3K/Akt) signaling pathway, which inhibits GC apoptosis and may help improve fertility (20). In PCOS mouse models, Akt expression was significantly increased in the astaxanthin treatment group (*P* < 0.05). Although Akt expression in the metformin group was lower than in the astaxanthin group, it was still significantly higher than that in the control group, and both groups promoted ovulation (46).

Clinical studies have also shown that after eight weeks of Administration of astaxanthin, the expression of DR5 gene and protein decreased (*p* < 0.05), while the gene expression of Caspase8 (*p* > 0.05) and BAX (*p* < 0.05) showed a downward trend, indicating that Administration of astaxanthin can regulate apoptosis-related factors and affect apoptosis pathways in GCs. Another study conducted by Masoome Jabarpour et al (47) included 58 PCOS patients who received 12 mg/day of astaxanthin for eight weeks. The results showed that compared with the control group, the expression of DR5 gene and protein in granulosa cells of the astaxanthin group significantly decreased. This study suggests that astaxanthin intervention improves the levels of apoptosis-related factors in patients’ serum and follicular fluid (FF) and modulates the expression of key genes and proteins in the granulosa cell apoptosis pathway (29, 48).

In summary, astaxanthin demonstrates potential therapeutic value in reducing cell apoptosis, improving follicular development, and enhancing ovarian morphology by regulating signaling pathways such as PI3K/Akt. These findings indicate that astaxanthin maintains follicular granulosa cell function through multiple signaling pathways, reduces abnormal apoptosis, and improves ovarian reserve and reproductive outcomes.

### 5.2 Improving oocyte quality and embryo outcomes

**Figure 2:**
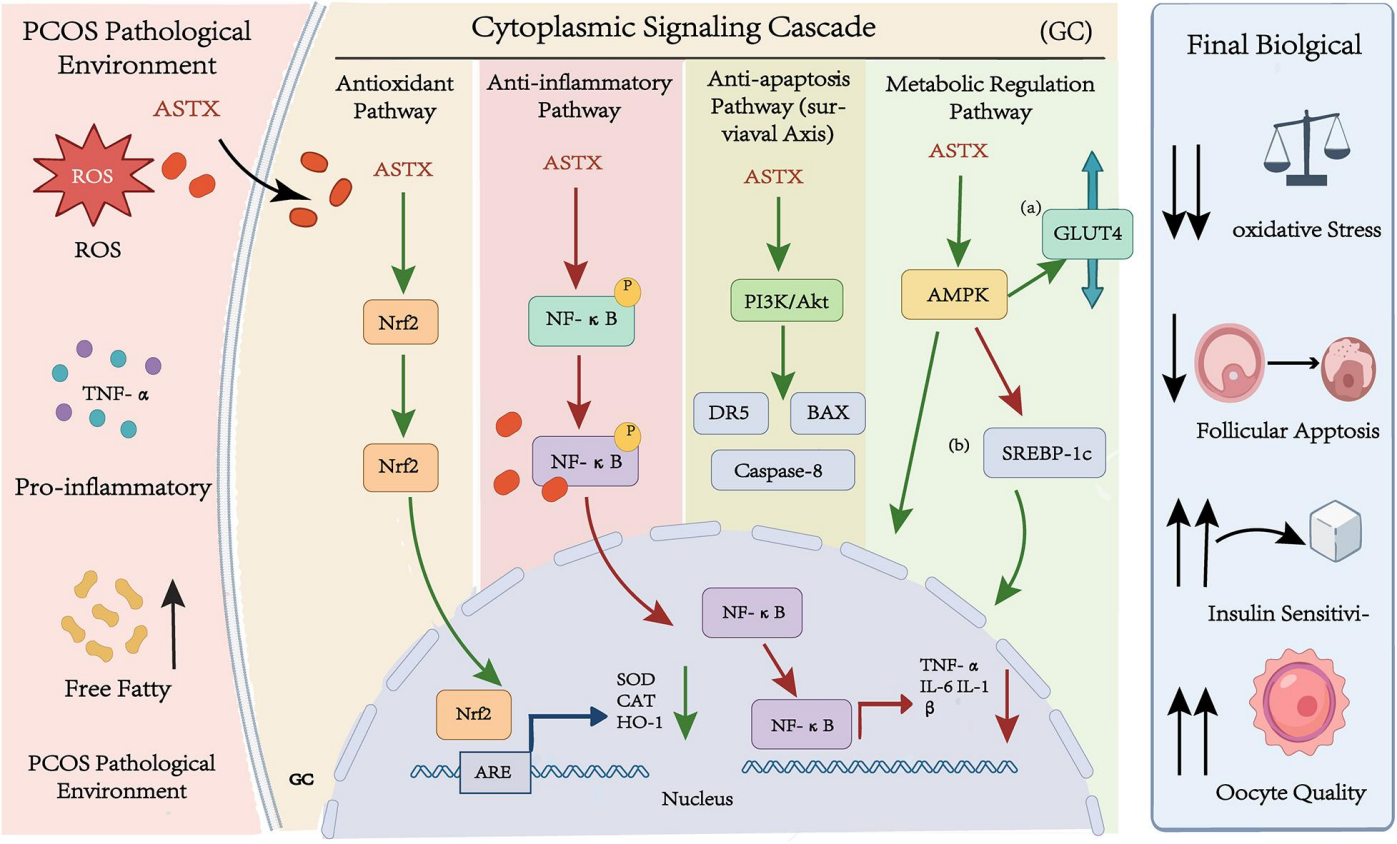
The signaling pathways and related molecules of astaxanthin in regulating PCOS.

The quality of oocytes affects the efficiency of assisted reproductive technologies (ART) such as *in vitro* fertilization (IVF), intracytoplasmic sperm injection (ICSI), and somatic cell nuclear transfer (SCNT), and is crucial for successful embryo development and subsequent implantation after transfer (49). Astaxanthin can improve oocyte quality and embryonic developmental outcomes in patients with PCOS through multiple pathways. Wen et al (50) demonstrated that supplementing astaxanthin in the culture medium effectively prevents post-ovulatory oocyte aging and prolongs the *in vitro* lifespan of oocytes. This mechanism involves the specific binding of astaxanthin to tumor necrosis factor receptor 2 (TNFR2) on the oocyte membrane, blocking the interaction between TNF-α and TNFR2, thereby inhibiting excessive activation of the downstream TNF signaling pathway, reducing inflammatory damage to oocytes, effectively extending oocyte survival *in vitro*, and enhancing their early embryonic developmental potential. Dujíčková et al. (51) reported that astaxanthin can effectively alleviate oxidative stress experienced by oocytes during *in vitro* maturation or vitrification and freezing processes by lowering reactive oxygen species levels, reducing lipid peroxidation, and improving mitochondrial membrane potential, thereby maintaining cellular energy metabolism homeostasis. Functionally, astaxanthin treatment increases oocyte maturation rates, embryonic formation rates, and embryo quality, upregulates the anti-apoptotic gene BCL2, downregulates the pro-apoptotic gene CAS9, and regulates the expression of key genes such as BCL2 and GPX4 to enhance embryo developmental potential. Yuan et al. (52) found that astaxanthin significantly improves the *in vitro* maturation rate of mouse oocytes and promotes subsequent early embryo development by regulating the expression of genes related to mitochondrial function and enhancing mitochondrial activity, demonstrating significant potential for application in assisted reproductive technologies. Through multiple mechanisms—including delaying cellular aging, alleviating oxidative stress, optimizing mitochondrial function, and regulating key gene expression—astaxanthin collectively improves oocyte quality and embryonic developmental outcomes in PCOS patients, showing promising application prospects in ART (as shown in Figure 2).

## 6 Safety and research outlook

Natural astaxanthin exhibits remarkable anti-inflammatory and antioxidant activities, showing promising application prospects in intervening the pathological mechanisms of PCOS. It has been reported that in 2014, the Panel on Dietetic Products, Nutrition and Allergies of the European Food Safety Authority (EFSA) conducted a safety assessment on astaxanthin-rich ingredients in AstaREAL preparations and issued a positive opinion. The assessment concluded that the consumption of such preparations poses no harm to nutritional health and carries no genotoxic safety risks (53). Meanwhile, the highly unsaturated molecular structure of astaxanthin makes it extremely vulnerable to damage during isolation, production and storage, which further exacerbates the challenges for its application (54).

The effects of astaxanthin are highly dependent on specific conditions. For example, research by Tana et al. (55) found that supplementing astaxanthin in fertilization culture medium significantly increased blastocyst formation rate and quality during *in vitro* fertilization, whereas supplementation during the embryo culture stage could potentially impede embryonic development. This indicates that its efficacy is highly dependent on timing and the local microenvironment, and it also reflects the current limited understanding of the specific mechanisms of astaxanthin at different stages of germ cell development.

Future research should focus on the following areas: First, large-scale, long-term randomized controlled trials are needed to clarify the long-term efficacy and safety of astaxanthin in PCOS patients. Given the poor solubility and stability of astaxanthin, which result in low oral bioavailability, technologies such as microencapsulation can enhance its performance by providing physical shielding against oxygen and light, reducing molecular mobility, and leveraging lipid or polymer interactions to achieve stabilization. These mechanisms collectively improve the chemical stability, functional properties, and oral bioavailability of this lipophilic bioactive compound (56, 57). Additionally, the synergistic effects of astaxanthin with other active compounds should be further explored. Recent studies have shown that combined use of astaxanthin and curcumin can synergistically reduce ovarian oxidative stress and chronic inflammation through multiple pathways, significantly improving ovarian reserve function and ovulation quality in PCOS patients. Moreover, combination strategies of astaxanthin with conventional drugs such as metformin offer new avenues for PCOS treatment.

Based on existing evidence, astaxanthin, as a natural dietary supplement, shows multi-targeted intervention advantages in the adjuvant treatment of PCOS, particularly in improving insulin sensitivity, regulating hormonal balance, and protecting ovarian function. Future studies should establish the optimal dosing strategies through more rigorous clinical research and promote its application in personalized treatment of PCOS.

## 7 Summary

Astaxanthin effectively intervenes in the core pathophysiological processes of PCOS through multiple molecular pathways, including activating the Nrf2 antioxidant pathway, inhibiting NF-κB inflammatory signaling, and regulating the PI3K/Akt apoptosis pathway. These interventions can alleviate oxidative stress, suppress chronic inflammation, improve insulin sensitivity, and protect ovarian function. As a potent antioxidant, astaxanthin shows significant potential in addressing the two core pathological mechanisms of PCOS—oxidative stress and chronic inflammation. It can enhance insulin sensitivity, modulate hormone levels, and improve oocyte and embryo quality. However, current studies are limited in scale, may involve overlapping samples, and the bioavailability and dose-response of astaxanthin have not been systematically evaluated. Future research should focus on rigorously designed, large-scale, long-term follow-up studies to validate its therapeutic potential and establish the optimal clinical dosing strategy. Overall, as a multitargeted natural compound, astaxanthin demonstrates unique advantages in improving the pathophysiological abnormalities of PCOS and holds promise as a new option for the comprehensive management of PCOS (as shown in Table 1).

**Table 1:** Mechanisms of astaxanthin supplementation in improving PCOS.

Mechanism of action	Signaling pathways	Changes in molecules/indicators	Detailed mechanisms	References
Antioxidant Stress	Nrf2/ARE; Nrf2/HO-1	Nrf2↑;HO-1↑;NQO1↑;SOD↑;CAT↑; GSH-Px↑; TAC↑; MDA↓;	1. Activates the Nrf2/ARE pathway to initiate the expression of downstream antioxidant enzymes. 2. Enhances cellular antioxidant defense via the Nrf2/HO-1 signaling pathway, and optimizes the ovarian microenvironment.	(16, 17, 19, 21, 23)
Anti-inflammatory Effects	NF-κB pathway; Endoplasmic reticulum stress-apoptosis pathway;Wnt/β-catenin; MEK/ERK pathway	TNF-α↓;IL-6↓; IL-1β↓;GRP78↓; CHOP↓;XBP1↓; Active caspase-3↓	1. Inhibits IκB-α degradation and NF-κB nuclear translocation, reducing the release of pro-inflammatory factors. 2. Alleviates endoplasmic reticulum stress and inhibits apoptosis mediated by factors like CHOP. 3. Upregulates Klotho expression and inhibits the Wnt/β-catenin and MEK/ERK signaling pathways, further suppressing inflammatory responses. 4. Regulates hypothalamic inflammation and restores the function of the hypothalamic-pituitary-ovarian (HPO) axis.	(24–26, 28–31)
Improving Insulin Resistance & Glucose Metabolism	AMPK pathway; PI3K/Akt; NF-κB/JAK2/STAT3 pathway; Insulin/IGF-1 signaling pathway	GLUT4↑; Fasting blood glucose↓; Fasting insulin↓; HOMA-IR index↓; S6K1 activity↓; TNF-α↓; IL-6↓	Activates the AMPK pathway to promote GLUT4 membrane translocation and improve glucose uptake. 2. Enhances insulin signal sensitivity via the PI3K/Akt pathway. 3. Regulates the NF-κB/JAK2/STAT3 pathway to alleviate inflammation-induced insulin resistance.	(29, 32–35)
Improving Lipid Metabolism	Nrf2/SREBP-1c pathway; Gut microbiota regulation; Insulin/IGF-1 signaling pathway	TC↓; TG↓; LDL-C↓; SCFAs↑; Hepatic lipid deposition↓; FASN expression↓	Activates the Nrf2 pathway and inhibits SREBP-1c and its downstream lipogenic genes (e.g., FASN). 2. Optimizes gut microbiota structure and increases short-chain fatty acids (SCFAs) production to improve lipid metabolism. 3. Reduces hepatic lipid accumulation and serum lipid levels.	(36–40, 45)
Ovarian Protection & Anti-apoptosis	PI3K/Akt pathway; Endoplasmic reticulum stress pathway; Nrf2/HO-1 pathway	p-Akt↑; Bcl-2↑/Bax↓;Caspase-3↓;Nrf2↑; HO-1↑	1. Activates the PI3K/Akt pathway to inhibit granulosa cell apoptosis and promote follicular development. 2. Alleviates endoplasmic reticulum stress to indirectly suppress cell apoptosis. 3. Enhances ovarian antioxidant defense via the Nrf2/HO-1 pathway to improve oocyte maturation rate and quality.	(20, 46–52)
